# Morgagni’s hernia in adult

**DOI:** 10.11604/pamj.2023.44.26.38571

**Published:** 2023-01-12

**Authors:** Soultana Foutzitzi, Savas Deftereos

**Affiliations:** 1 Radiology Department, Democritus University of Thrace, Alexandroupolis, Greece

**Keywords:** Hernia, Morgagni, congenital diaphragmatic hernia

## Image in medicine

A 67-year-old woman presented in our radiology department for a preoperative check-up. From her medical history, a left breast lumpectomy was referred. In chest X-ray the lesion contains at least two small air-filled areas (A). The differential diagnoses include abscess, pericardial cyst, Morgagni´s hernia, etc. On followed CT a large Morgagni hernia was revealed (B,C,D). Herniation occurs through the Morgagni´s foramen adjacent to the xiphoid process and contains omentum and transverse colon while part of the stomach tends towards the hernia (C,D). Morgagni's hernia is a rare congenital diaphragmatic hernia (CDH), usually identified in childhood and occasionally in adults (first described in 1769 by Giovanni Battista Morgagni, an Italian anatomist). This defect is located in the anterior diaphragm between the sternal xiphoid process and the costochondral attachments of the diaphragm. Morgagni's hernia constitutes 2%-5% of all CDH, with approximately 90% affecting the right side, 5% the left side, and 5% being bilateral. In adults, protrusion of omentum is common, and only rarely (as in this present case) with bowel, stomach, or liver. A recent review of the literature found that Morgagni hernias are more frequently detected in women (61%) with an average age of 58 years. Overall, 91% of hernias were found on the right (Right > left (heart protects)). When symptomatic, complaints relate to pulmonary or bowel dysfunction or chest pain. Predisposing conditions are similar to those for other abdominal hernias and include pregnancy, trauma, obesity, chronic constipation, and chronic cough.

**Figure 1:**
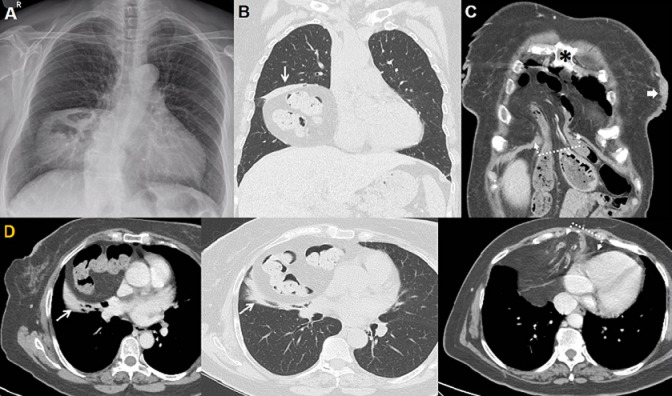
A) chest X-ray: well-defined heterogenous mass with presence of air adjacent to right heart aspect, between diaphragm and minor (horizontal) fissure; scoliosis of the spine is also noted; B, C) CT reconstruction in coronal plane (lung and mediastinal window) revealed bowel loops herniated from foramen of Morgagni (note the para-retrosternal position-black star- of bowel loops which insert from a diaphragmatic foramen -dot line); focal thickening of the left breast skin (arrow), consisted with the known cancer; D) the same findings in axial views in lung (middle view) and in mediastinal window; thin arrows (including B view): compressive atelectasis

